# Urban-Rural County and State Differences in Chronic Obstructive Pulmonary Disease — United States, 2015

**DOI:** 10.15585/mmwr.mm6707a1

**Published:** 2018-02-23

**Authors:** Janet B. Croft, Anne G. Wheaton, Yong Liu, Fang Xu, Hua Lu, Kevin A. Matthews, Timothy J. Cunningham, Yan Wang, James B. Holt

**Affiliations:** 1 Division of Population Health, National Center for Chronic Disease Prevention and Health Promotion, CDC.

Chronic obstructive pulmonary disease (COPD) accounts for the majority of deaths from chronic lower respiratory diseases, the third leading cause of death in the United States in 2015 and the fourth leading cause in 2016.* Major risk factors include tobacco exposure, occupational and environmental exposures, respiratory infections, and genetics.^†^ State variations in COPD outcomes (*1*) suggest that it might be more common in states with large rural areas. To assess urban-rural variations in COPD prevalence, hospitalizations, and mortality; obtain county-level estimates; and update state-level variations in COPD measures, CDC analyzed 2015 data from the Behavioral Risk Factor Surveillance System (BRFSS), Medicare hospital records, and death certificate data from the National Vital Statistics System (NVSS). Overall, 15.5 million adults aged ≥18 years (5.9% age-adjusted prevalence) reported ever receiving a diagnosis of COPD; there were approximately 335,000 Medicare hospitalizations (11.5 per 1,000 Medicare enrollees aged ≥65 years) and 150,350 deaths in which COPD was listed as the underlying cause for persons of all ages (40.3 per 100,000 population). COPD prevalence, Medicare hospitalizations, and deaths were significantly higher among persons living in rural areas than among those living in micropolitan or metropolitan areas. Among seven states in the highest quartile for all three measures, Arkansas, Kentucky, Mississippi, and West Virginia were also in the upper quartile (≥18%) for rural residents. Overcoming barriers to prevention, early diagnosis, treatment, and management of COPD with primary care provider education, Internet access, physical activity and self-management programs, and improved access to pulmonary rehabilitation and oxygen therapy are needed to improve quality of life and reduce COPD mortality.

The National Center for Health Statistics (NCHS) 2013 Urban-Rural Classification Scheme for Counties, which uses 2010 U.S. Census population data and the February 2013 Office of Management and Budget designations of metropolitan statistical area, micropolitan statistical area, or noncore area (*2*), was used to classify urban-rural status of BRFSS respondents, Medicare inpatient claims, decedents, and populations at risk based on reported county of residence. The six categories include large central metropolitan, large fringe metropolitan, medium metropolitan, small metropolitan, micropolitan, and noncore (rural). Definitions and use of these categories have been described previously (*2*,*3*).

Prevalence of diagnosed COPD was estimated using the 2015 BRFSS survey, an annual state-based, random-digit–dialed cellular and landline telephone survey of the noninstitutionalized U.S. population aged ≥18 years^§^ that is conducted by state health departments in collaboration with CDC. In 2015, the median survey response rate for the 50 states and District of Columbia (DC) was 46.6% and ranged from 33.9% to 61.1%.^¶^ Diagnosed COPD was defined as an affirmative response to the question “Has a doctor, nurse, or other health professional ever told you that you had chronic obstructive pulmonary disease or COPD, emphysema, or chronic bronchitis?” State analyses included 426,838 (98.3%) respondents in the 50 states and DC after exclusions for missing information on COPD or age (Table 1). Urban-rural analyses included 426,736 (98.2%) respondents after excluding those who had missing information for COPD, age, or county code.

A multilevel regression and poststratification approach (*4*) was used to estimate model-predicted COPD prevalence for U.S. counties in 2015. High internal validity was determined by comparing modeled estimates with actual unweighted BRFSS survey estimates in 1,507 counties with ≥50 respondents (Pearson correlation coefficient = 0.68; p<0.001), and with weighted BRFSS survey estimates in 195 counties with ≥500 respondents and relative standard errors <0.30 (Pearson correlation coefficient = 0.74; p<0.001).

Medicare enrollment records and data from 100% of Part A (inpatient hospital) claims in 2015 were obtained from the Centers for Medicare & Medicaid Services. Analyses were limited to 30,212,024 living Medicare Part A enrollees aged ≥65 years who were eligible for fee-for-service hospitalizations on July 1, 2015, and all 335,362 fee-for-service inpatient hospital claims with a first-listed diagnosis of COPD that were submitted in 2015 for Medicare Part A enrollees aged ≥65 years. COPD was defined by *International Classification of Diseases, Ninth Edition, Clinical Modification* (ICD-9-CM) codes 490–492 or 496 or ICD-10-CM codes J40–J44.** Urban-rural analyses were limited to 335,102 (99.9%) hospital claims.

Mortality data for all ages were analyzed using CDC WONDER, an interactive public-use Web-based tool.^††^ CDC WONDER mortality data from NVSS contain information from all resident death certificates filed in the 50 states and DC. CDC WONDER queries generated numbers of deaths, age-adjusted death rates, 95% confidence intervals (CIs), and population denominators for groups defined by state and the 2013 NCHS urban-rural classification of decedents. Deaths caused by COPD were defined by ICD-10 codes J40–J44, in which COPD was the underlying cause of death on the death certificate. CDC also obtained population estimates for 2015 from CDC WONDER to calculate the percentage of U.S. and state residents who lived in a rural county as classified by the NCHS 2013 urban-rural county classification.

Age-adjusted prevalence of diagnosed COPD for persons aged ≥18 years, Medicare hospitalization rate for persons aged ≥65 years, death rate for all ages, and 95% CI for each estimate were calculated by urban-rural classification and state. For BRFSS analyses, statistical software was used to account for the complex sampling design. Differences in COPD prevalence among rural respondents compared with those of other urban-rural subgroups were determined by t-tests. Urban-rural differences in Medicare hospitalizations and death rates were determined by the Z-test. All two-sided tests were considered statistically significant at α = 0.05.

In 2015, approximately 15.5 million adults aged ≥18 years (unadjusted prevalence = 6.3% and age-adjusted prevalence = 5.9%) had self-reported diagnosed COPD. County-level estimates of COPD prevalence ranged from 3.2% to 15.6% (Figure). U.S. counties within the highest quartile of county-level estimates (8.5%−15.6%) tended to be located in nonmetropolitan areas of Alabama, Arizona, Arkansas, Georgia, Kentucky, Maine, Michigan, Missouri, Ohio, Oklahoma, Tennessee, and West Virginia (Figure).

**FIGURE:**
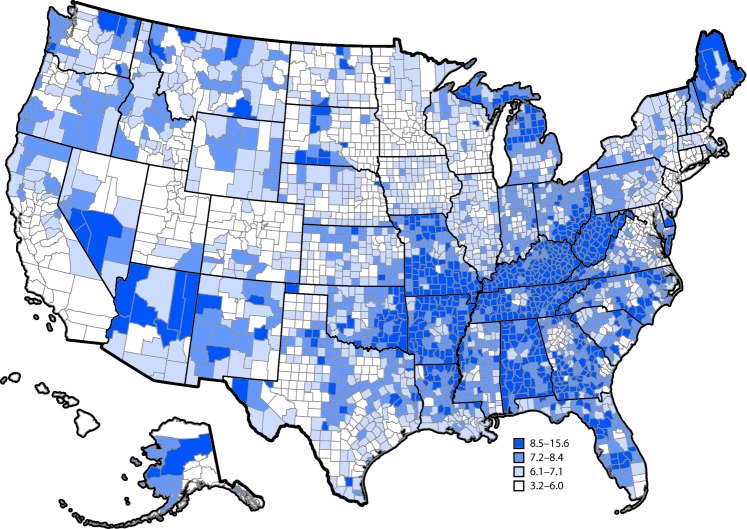
Unadjusted prevalence of diagnosed chronic obstructive pulmonary disease among adults aged ≥18 years, by county — United States, 2015

Age-adjusted prevalence of diagnosed COPD among adults aged ≥18 years increased with less urbanicity from 4.7% among populations living in large metropolitan centers to 8.2% among adults living in rural areas (Table 1). Medicare hospitalizations (per 1,000 enrollees) for COPD were 11.4 among enrollees aged ≥65 years living in large metropolitan centers and 13.8 among those living in rural areas. Age-adjusted death rates (per 100,000 population) for COPD as the underlying cause also increased with less urbanicity from 32.0 for U.S. residents living in large metropolitan centers to 54.5 for those living in rural areas. There was a consistent pattern for significantly higher estimates of COPD measures from all three independent data systems among adults living in rural areas than among those living in micropolitan or metropolitan areas.

**TABLE 1:** Age-adjusted estimates of selected COPD measures, by urban-rural status of county* — United States, 2015

COPD measure	Overall^†^	Large metropolitan center	Large fringe metropolitan	Medium metropolitan	Small metropolitan	Micropolitan	Noncore (rural)
**Adult prevalence** ^§^							
BRFSS respondents	426,838	69,442	81,788	92,571	57,415	65,029	60,491
Estimated no. in population (rounded to 1,000s) with diagnosed COPD	15,460,000	3,566,000	3,406,000	3,452,000	1,661,000	1,796,000	1,576,000
% (95% CI)	5.9 (5.8−6.0)	4.7 (4.5−5.0)	5.3 (5.0−5.5)	6.4 (6.2−6.7)	7.0 (6.6−7.3)	7.6 (7.2−8.0)	8.2 (7.8−8.7)
**Medicare hospitalizations** ^¶^							
Number of Medicare enrollees, aged ≥65 years, in fee-for-service plan	30,212,024	6,812,852	7,402,029	6,510,167	3,361,075	3,400,705	2,701,592
Hospital claims with COPD as first-listed diagnosis	335,362	74,616	78,220	68,291	35,798	41,653	36,524
Rate per 1,000 (95% CI)	11.5 (11.4−11.5)	11.4 (11.3−11.5)	11.0 (11.0−11.1)	10.8 (10.7−10.9)	10.9 (10.8−11.0)	12.5 (12.4−12.6)	13.8 (13.6−13.9)
Deaths**							
U.S. population (all ages)	321,418,820	98,997,449	79,867,097	67,041,154	29,346,517	27,260,617	18,905,986
Number of deaths with COPD as underlying cause	150,350	32,309	32,718	33,619	17,419	19,019	15,266
Rate per 100,000 (95% CI)	40.3 (40.1−40.5)	32.0 (31.6−32.3)	36.2 (35.8−36.6)	41.9 (41.5−42.4)	47.0 (46.3−47.7)	52.8 (52.1−53.6)	54.5 (53.6−55.4)

**Abbreviations:** BRFSS = Behavioral Risk Factor Surveillance System; CI = confidence interval; COPD = chronic obstructive pulmonary disease (includes emphysema and chronic bronchitis).

* As defined in the National Center for Health Statistics 2013 Urban-Rural Classification Scheme for Counties.

^†^ Numbers in urban-rural categories for prevalence and Medicare hospitalizations do not sum to the overall number because 0.02% of eligible BRFSS respondents, 0.08% of eligible Medicare enrollees, and 0.08% of COPD Medicare claims could not be assigned an urban-rural classification.

^§^ Percentage ever told by a doctor, nurse, or other health professional that respondent had COPD, emphysema, or chronic bronchitis among adults aged ≥18 years in the 2015 Behavioral Risk Factor Surveillance System survey. Age-adjusted to the 2000 U.S. projected population, aged ≥18 years, using five age groups (18–44, 45–54, 55–64, 65–74, and ≥75 years).

^¶^ Hospitalizations among adults aged ≥65 years with a first-listed diagnosis claim for COPD *International Classification of Diseases, Ninth Revision, Clinical Modification* (ICD-9-CM) codes 490–492, or 496 or ICD-10-CM codes J40–J44 in the 2015 Medicare Part A hospital claims records. Hospital rates per 1,000 Medicare fee-for-service enrollees aged ≥65 years were age-adjusted to the 2000 U.S. projected population aged ≥65 years, using two age groups (65–74 and ≥75 years).

** Death rate per 100,000 U.S. population (including children) for COPD (ICD-10 codes J40–J44) reported as the underlying cause of death on the death certificate; age-adjusted to the total 2000 U.S. projected population, using 11 age groups (<1, 1–4, 5–14, 15–24, 25–34, 35–44, 45–54, 55–64, 65–74, 75–84, and ≥85 years).

Overall 5.9% of U.S. residents lived in rural counties in 2015. State-specific percentages of rural residents ranged from zero percent in Connecticut, Delaware, District of Columbia, New Jersey, and Rhode Island to 34.7% in Montana (Table 2). State-specific age-adjusted prevalence of COPD among adults aged ≥18 years in 2015 ranged from 3.8% in Utah to 12.0% in West Virginia. State-specific age-adjusted Medicare hospitalization rates (per 1,000 enrollees) among enrollees aged ≥65 years ranged from 3.7 in Utah to 19.7 in West Virginia. State-specific age-adjusted death rates (per 100,000 population) in 2015 ranged from 15.8 in Hawaii to 64.3 in Oklahoma. Of the seven states (Alabama, Arkansas, Indiana, Kentucky, Mississippi, Tennessee, and West Virginia) that were in the highest quartiles for all three measures in 2015, four states (Arkansas, Kentucky, Mississippi, and West Virginia) were also in the highest quartile (≥18%) for percentage of rural residents.

**TABLE 2:** Percentage of rural residents and age-adjusted estimates of selected COPD measures, by state — United States, 2015

State	% rural residents*	Rank order in % rural residents	No. in U.S. population with COPD^†^	% (95% CI)^§^	No. of Medicare hospitalizations^¶^	Rate per 1,000 (95% CI)^¶^	No. of deaths	Rate per 100,000 (95% CI)**
Alabama	12.8	16	393,000	9.9 (9.0−10.9)	7,691	14.3 (14.0−14.6)	3,217	55.2 (53.3−57.1)
Alaska	26.1	5	22,000	4.1 (3.3−5.1)	380	6.3 (5.6−6.9)	193	36.1 (30.7−41.6)
Arizona	1.5	38	325,000	5.8 (5.2−6.5)	4,711	8.3 (8.1−8.5)	3,570	42.4 (41.0−43.8)
Arkansas	19.1	11	219,000	9.1 (8.0−10.5)	4,806	13.3 (12.9−13.7)	2,234	61.3 (58.7−63.8)
California	0.7	41	1,207,000	4.0 (3.6−4.4)	20,289	7.9 (7.8−8.1)	13,092	31.8 (31.3−32.4)
Colorado	5.6	26	179,000	4.2 (3.8−4.6)	2,376	6.4 (6.1−6.6)	2,514	46.6 (44.8−48.5)
Connecticut	0.0	43	143,000	4.6 (4.1−5.1)	3,798	9.7 (9.4−10.0)	1,309	28.4 (26.8−30.0)
Delaware	0.0	43	51,000	6.3 (5.3−7.5)	1,137	8.6 (8.1−9.1)	494	40.9 (37.3−44.6)
DC	0.0	43	28,000	5.9 (4.9−7.2)	445	7.5 (6.8−8.2)	134	21.5 (17.8−25.2)
Florida	1.7	37	1,117,000	6.0 (5.4−6.6)	32,274	15.9 (15.7−16.1)	11,461	37.4 (36.7−38.1)
Georgia	7.7	22	532,000	6.7 (6.0−7.6)	9.425	11.9 (11.7−12.2)	4,501	45.7 (44.3−47.1)
Hawaii	0.0	43	48,000	4.1 (3.5−4.9)	663	6.2 (5.7−6.7)	303	15.8 (14.0−17.6)
Idaho	8.3	21	59,000	4.5 (3.9−5.3)	942	6.3 (5.9−6.7)	817	44.8 (41.7−47.9)
Illinois	4.7	29	568,000	5.4 (4.7−6.3)	14,964	11.4 (11.2−11.6)	5,360	36.8 (35.8−37.8)
Indiana	7.0	23	400,000	7.4 (6.6−8.3)	9,048	13.1 (12.9−13.4)	4,096	53.7 (52.1−55.4)
Iowa	25.2	7	136,000	5.2 (4.6−6.0)	3,407	8.3 (8.0−8.6)	1,949	47.5 (45.4−49.7)
Kansas	13.5	15	134,000	5.8 (5.5−6.2)	2,764	8.0 (7.7−8.3)	1,665	48.5 (46.1−50.8)
Kentucky	22.3	8	410,000	11.2 (10.2−12.3)	8,618	19.1 (18.7−19.5)	3,280	63.2 (61.1−65.4)
Louisiana	7.7	22	265,000	7.1 (6.3−8.0)	5,452	13.5 (13.2−13.9)	2,125	42.1 (40.3−43.9)
Maine	31.8	2	86,000	7.0 (6.3−7.8)	1,986	11.3 (10.8−11.8)	1,003	52.5 (49.2−55.8)
Maryland	1.4	39	282,000	5.8 (5.1−6.5)	5,841	8.4 (8.2−8.6)	1,945	29.2 (27.9−30.5)
Massachusetts	0.2	42	303,000	5.3 (4.8−6.0)	8,566	11.4 (11.2−11.7)	2,668	31.6 (30.4−32.8)
Michigan	6.7	24	584,000	6.9 (6.3−7.6)	13,338	13.9 (13.7−14.1)	5,700	46.2 (45.0−47.4)
Minnesota	10.5	18	187,000	4.2 (3.8−4.5)	3,910	12.7 (12.3−13.1)	2,273	35.1 (33.7−36.6)
Mississippi	22.2	9	173,000	7.2 (6.4−8.2)	5,040	14.3 (13.9−14.7)	1,865	55.3 (52.8−57.8)
Missouri	13.7	14	387,000	7.9 (7.1−8.9)	7,587	12.2 (11.9−12.5)	3,843	51.4 (49.8−53.1)
Montana	34.7	1	45,000	5.0 (4.3−5.8)	918	7.0 (6.5−7.4)	663	48.8 (45.0−52.5)
Nebraska	18.0	12	77,000	5.0 (4.6−5.5)	2,061	8.9 (8.5−9.3)	1,127	50.0 (47.1−53.0)
Nevada	1.1	40	145,000	6.2 (5.1−7.6)	2,079	9.0 (8.6−9.4)	1,591	53.2 (50.5−55.8)
New Hampshire	3.6	32	70,000	6.1 (5.3−6.9)	1,794	9.5 (9.0−9.9)	681	40.3 (37.3−43.4)
New Jersey	0.0	43	341,000	4.6 (4.1−5.1)	10,454	10.1 (9.9−10.3)	3,057	28.2 (27.1−29.2)
New Mexico	4.4	30	94,000	5.5 (4.9−6.3)	1,530	8.1 (7.7−8.6)	1,079	43.4 (40.8−46.0)
New York	2.0	36	882,000	5.3 (4.8−5.8)	20,489	12.3 (12.2−12.5)	6,755	28.3 (27.6−29.0)
North Carolina	6.3	25	573,000	7.0 (6.3−7.7)	10,632	11.2 (11.0−11.4)	5,077	44.1 (42.9−45.3)
North Dakota	26.5	4	30,000	4.8 (4.2−5.6)	695	8.4 (7.8−9.0)	340	38.7 (34.5−42.9)
Ohio	3.9	31	705,000	7.1 (6.5−7.9)	16,189	16.7 (16.4−16.9)	7,000	48.0 (46.9−49.1)
Oklahoma	13.9	13	255,000	8.2 (7.4−9.1)	5,563	12.6 (12.3−12.9)	2,863	64.3 (61.9−66.7)
Oregon	2.4	34	174,000	5.1 (4.5−5.8)	2,442	7.6 (7.3−7.9)	2,037	40.7 (38.9−42.5)
Pennsylvania	3.2	33	701,000	6.2 (5.5−7.0)	17,795	14.9 (14.7−15.2)	6,457	36.7 (35.8−37.6)
Rhode Island	0.0	43	52,000	5.7 (4.9−6.5)	1,435	15.2 (14.4−16.0)	498	35.8 (32.6−39.0)
South Carolina	6.3	25	272,000	6.7 (6.1−7.3)	5,666	10.0 (9.7−10.2)	2,828	48.5 (46.6−50.3)
South Dakota	25.4	6	36,000	5.2 (4.4−6.1)	976	9.4 (8.8−10.0)	488	44.0 (40.0−47.9)
Tennessee	9.8	19	486,000	8.9 (8.0−10.0)	9,875	15.7 (15.3−16.0)	4,151	53.7 (52.1−55.4)
Texas	5.1	27	1,032,000	5.1 (4.6−5.7)	22,975	11.7 (11.5−11.9)	9,939	40.2 (39.4−41.0)
Utah	4.8	28	75,000	3.8 (3.4−4.3)	683	3.7 (3.4−4.0)	770	32.3 (30.0−34.6)
Vermont	26.1	5	31,000	5.6 (4.9−6.3)	660	6.9 (6.4−7.5)	345	41.0 (36.6−45.4)
Virginia	9.3	20	374,000	5.5 (5.0−6.0)	7,248	8.1 (7.9−8.2)	3,258	35.8 (34.6−37.1)
Washington	2.2	35	335,000	5.8 (5.3−6.3)	3,608	5.4 (5.3−5.6)	3,016	37.9 (36.5−39.3)
West Virginia	21.9	10	194,000	12.0 (11.1−13.0)	4,388	19.7 (19.1−20.2)	1,597	63.1 (60.0−66.3)
Wisconsin	12.5	17	209,000	4.2 (3.6−4.8)	5,179	10.3 (10.0−10.6)	2,761	38.1 (36.6−39.5)
Wyoming	27.4	3	32,000	6.8 (5.9−7.9)	570	7.7 (7.1−8.4)	361	55.9 (50.0−61.7)
**50 states and DC**	**5.9**	**—**	**15,460,000**	**5.9 (5.8−6.0)**	**335,362**	**11.5 (11.4−11.5)**	**150,350**	**40.3 (40.1−40.5)**

**Abbreviations:** BRFSS = Behavioral Risk Factor Surveillance System; CI = confidence interval; COPD = chronic obstructive pulmonary disease (includes emphysema and chronic bronchitis); DC = District of Columbia.

*Percentages of residents who live in rural (noncore) counties were calculated from 2015 bridged-race postcensal estimates (July 1, 2015) for populations that were defined by the 2013 National Center for Health Statistics 2013 Urban-Rural Classification Scheme for Counties and obtained from CDC WONDER.

^†^ Estimated number of adults with diagnosed COPD rounded to 1,000s.

^§^ Percentage ever told by a doctor, nurse, or other health professional that respondent had COPD, emphysema, or chronic bronchitis among adults aged ≥18 years in the 2015 BRFSS survey. Age-adjusted to the 2000 U.S. projected population, aged ≥18 years, using five age groups (18–44, 45–54, 55–64, 65–74, and ≥75 years).

^¶^ Hospitalizations among adults aged ≥65 years with a first-listed diagnosis claim for COPD *International Classification of Diseases, Ninth Revision, Clinical Modification* (ICD-9-CM) codes 490–492, or 496 or ICD-10-CM codes J40–J44 in the 2015 Medicare Part A hospital claims records. Hospital rates per 1,000 Medicare fee-for-service enrollees aged ≥65 years were age-adjusted to the 2000 U.S. projected population aged ≥65 years, using two age groups (65–74 and ≥75 years).

** Death rate per 100,000 U.S. population (including children) for COPD (ICD-10 codes J40–J44) reported as the underlying cause of death on the death certificate. Age-adjusted to the total 2000 U.S. projected population, using 11 age groups (<1, 1–4, 5–14, 15–24, 25–34, 35–44, 45–54, 55–64, 65–74, 75–84, and ≥85 years).

## Discussion

In 2015, rural U.S. residents experienced higher age-adjusted COPD prevalence, Medicare hospitalizations for COPD as the first-listed diagnosis, and deaths caused by COPD than did residents in micropolitan or metropolitan areas. In addition to the major risk factors for COPD, which include tobacco smoke, environmental and occupational exposures, respiratory infections, and genetics, correlates include older ages, low socioeconomic status, and asthma history (*5*,*6*). Rural populations might have higher COPD risk because these populations have a greater proportion with a history of smoking (*3*), more secondhand smoke exposure but less access to smoking cessation programs,^§§^ and higher proportions of uninsured or lower socioeconomic residents, which might have limited access to early diagnosis, treatment, and management of COPD.^¶¶^ Rural respiratory exposures might include mold spores, organic toxic dust, and nitrogen dioxide, which are associated with COPD risk (*7*).

COPD management includes efforts to slow declining lung function, improve exercise tolerance, and prevent and treat exacerbations. Treatments include pulmonary rehabilitation, oxygen therapy, and medications. Smoking cessation programs, routine influenza and pneumococcal vaccinations, regular physical activity, and reductions in occupational and environmental exposures are also important. Barriers to health care in rural areas include cultural perceptions about seeking care, travel distance, absence of services, and financial burden (*8*). Access to early diagnosis, prompt treatment, and management of COPD by a pulmonologist is difficult for rural adults with COPD because of limited geographic accessibility to this COPD specialty (*9*). Therefore, much of the COPD in rural areas is diagnosed and managed by primary care providers (*9*). Level of care and patient-physician communication might vary, given that 27% of adults with COPD symptoms in 2016 reported that they had not talked with their physician about these symptoms (*10*). In a primary care physician survey, 71% said that they would use spirometry to assess patients with COPD symptoms, but they also reported that important barriers to diagnosing COPD included patient failure to report COPD symptoms or smoking history, poor treatment adherence, more immediate competing health issues, and diagnostic procedure costs (*10*). Whereas 68% of primary care physicians were aware that pulmonary rehabilitation programs were available to their patients, only 38% routinely prescribed this therapy for COPD patients (*10*). However, rural areas might have limited availability to these programs. Provision of online health care services (i.e., telemedicine) in rural areas could reduce some of these barriers by providing health education and support websites to patients and caregivers, appointment assistance, and ability to check assessment results online; however, lack of Internet access is still a barrier in some rural populations (*8*).

The findings in this report are subject to at least eight limitations. First, self-reported diagnosed COPD in BRFSS cannot be validated with medical records and might be subject to recall and social desirability biases; however, urban-rural variations in prevalence were similar to Medicare claims. Second, the BRFSS study population does not include adults who live in long-term care facilities, prisons, and other facilities; thus, findings are not generalizable to those populations. Third, state BRFSS response rates were relatively low, and response rates cannot be obtained by urban-rural classification. This might have resulted in overestimates or underestimates of COPD prevalence; however, a strength is that BRFSS provides large, stable sample sizes for all six urban-rural classifications. Fourth, the assumption that the six urban-rural classifications reflect consistent types of distinct populations and social environments within and across each state could potentially be incorrect. Fifth, county-level estimates are modeled and based on population characteristics such as distributions of older adults in the county; furthermore, it is not known how previous or current local interventions (e.g., tobacco cessation policies and programs) might have affected current COPD prevalence. Sixth, Medicare claims should not be interpreted as unique prevalent cases because some might reflect readmissions; however, these COPD estimates do reflect the actual Medicare burden for hospital facilities, pulmonary rehabilitation services, health care providers, caregivers, and other resources. Seventh, both Medicare hospital claims and death certificates might be subject to reporting preferences for certain diseases as the first-listed or underlying cause if there is a consistent regional or urban-rural preference. Finally, although the data reported here show higher COPD hospitalization and death rates for rural populations, they do not assess whether hospitalization and death rates among patients with COPD vary by urbanicity. 

Higher burdens of COPD among rural U.S. residents highlight needs for continued tobacco cessation programs and policies to prevent COPD and improve pulmonary function among smokers. Known barriers to care in rural areas suggest a need for improved access for adults with COPD to treatment strategies (pulmonary rehabilitation and oxygen therapy) and comprehensive chronic disease self-management programs. Health care providers and community partners who serve rural residents can help adults with COPD increase access to and participation in health care interventions. Federal agencies are promoting collaborative and coordinated efforts to educate the public, providers, patients, and caregivers about COPD and improve the prevention, diagnosis, and treatment of COPD. The COPD National Action Plan*** includes goals to expand access to online communities, develop clinical decision tools for primary health care providers, and conduct research to improve access to care for COPD in hard-to-reach areas. Promoting these efforts has the potential to improve quality of life for COPD patients and reduce hospital readmissions and COPD mortality.

**Summary**

**What is already known about this topic?**

Chronic obstructive pulmonary disease (COPD) is a leading cause of death and has been diagnosed in 15.5 million adults in 2015 in the United States. Risk factors include tobacco exposure, occupational and environmental exposures, respiratory infections, and genetics.

**What is added by this report?**

In 2015, rural U.S. residents had higher age-adjusted prevalence of COPD, of Medicare hospitalizations, and deaths caused by COPD than did residents living in micropolitan or metropolitan areas. Several states with the highest percentages of rural populations also had the highest estimates for all three measures.

**What are the implications for public health practice?**

Additional efforts are needed to prevent risk factors and overcome barriers to early diagnosis, and the appropriate treatment and management of COPD. Improving access to such health care might improve quality of life and reduce hospital readmissions among COPD patients and reduce COPD mortality.

## Footnotes

* Leading causes of death reported for 2015 at https://www.cdc.gov/nchs/data/nvsr/nvsr66/nvsr66_05.pdf and for 2016 at https://www.cdc.gov/nchs/products/databriefs/db293.htm.

†
https://www.cdc.gov/copd .

§
https://www.cdc.gov/brfss/annual_data/annual_2015.html .

¶ Response rates for BRFSS are calculated using standards set by American Association for Public Opinion Research response rate formula 4. The response rate is the number of respondents who completed the survey as a proportion of all eligible and likely eligible persons. http://www.aapor.org/AAPOR_Main/media/publications/Standard-Definitions20169theditionfinal.pdf. Response rates in 2015 for individual states are available at https://www.cdc.gov/brfss/annual_data/2015/pdf/2015-sdqr.pdf.

** *International Classification of Diseases, Ninth Edition, Clinical Modification* (ICD-9-CM) codes for COPD include 490 (bronchitis, not specified as acute or chronic), 491 (chronic bronchitis), 492 (emphysema), or 496 (chronic airway obstruction); ICD-10-CM codes include J40 (bronchitis, not specified as acute or chronic), J41 (simple and mucopurulent chronic bronchitis), J42 (unspecified chronic bronchitis), J43 (emphysema), or J44 (other COPD).

†† https://wonder.cdc.gov. Population estimates for groups defined by urban-rural status and state are bridged-race estimates of the July 1, 2015, resident population from the Vintage 2015 postcensal series that were released on June 28, 2016.

§§ http://www.lung.org/assets/documents/research/cutting-tobaccos-rural-roots.pdf.

¶¶
http://www.countyhealthrankings.org/reports/key-findings-2016 .

*** https://www.nhlbi.nih.gov/health-pro/resources/lung/copd-national-action-plan.
